# Phase transformations and vibrational properties of hybrid organic–inorganic perovskite MAPbI_3_ bulk at high pressure

**DOI:** 10.1038/s41598-023-43020-1

**Published:** 2023-10-06

**Authors:** Ardimas, Teerachote Pakornchote, Wiwittawin Sukmas, Sojiphong Chatraphorn, Stewart J. Clark, Thiti Bovornratanaraks

**Affiliations:** 1 Department of Nanoscience and Technology, Graduate School, Chulalongkorn University, Bangkok, 10330 Thailand; 2 Extreme Conditions Physics Research Laboratory (ECPRL) and Center of Excellence in Physics of Energy Materials (CE:PEM), Department of Physics, Faculty of Science, Chulalongkorn University, Bangkok, 10330 Thailand; 3 Thailand Center of Excellence in Physics, Commission on Higher Education, 328 Si Ayutthaya Road, Bangkok, 10400 Thailand; 4 Metallurgy and Materials Science Research Institute, Chulalongkorn University, Soi Chula 12, Phayathai Rd., Pathumwan, Bangkok, 10330 Thailand; 5 Department of Physics, Faculty of Science, Durham University, Durham, DH1 3LE UK

**Keywords:** Materials science, Physics

## Abstract

The structural stability and internal properties of hybrid organic–inorganic perovskites (HOIPs) have been widely investigated over the past few years. The interplay between organic cations and inorganic framework is one of the prominent features. Herein we report the evolution of Raman modes under pressure in the hybrid organic–inorganic perovskite MAPbI$_3$ by combining the experimental approach with the first-principles calculations. A bulk MAPbI$_3$ single crystal was synthesized via inverse temperature crystallization (ITC) technique and characterized by Raman spectroscopy, while the diamond anvil cells (DACs) was employed to compress the sample. The classification and behaviours of their Raman modes are presented. At ambient pressure, the vibrations of inorganic PbI$_6$ octahedra and organic MA dominate at a low-frequency range (60–760 cm$^{-1}$) and a fingerprint range (900–1500 cm$^{-1}$), respectively. The applied pressure exhibits two significant changes in the Raman spectrum and indicates of phase transition. The results obtained from both experiment and calculations of the second phase at 3.26 GPa reveal that the internal vibration intensity of the PbI$_6$ octahedra (< 110 cm$^{-1}$) reduces as absences of MA libration (150–270 cm$^{-1}$) and internal vibration of MA (450–750 cm$^{-1}$). Furthermore, the hydrogen interactions around 1300 cm$^{-1}$ remain strong high pressure up to 5.34 GPa.

## Introduction

Thanks to their high potential of absorbing sunlight, ease of fabrication, inexpensiveness, and long carrier diffusion length, the hybrid organic–inorganic perovskites (HOIPs) have become a promising research topic in various photovoltaic and optoelectronic applications, resulting in over 3000 publications so far^1^. The common HOIPs structure is written as ABX$_3$, wherein A, B, and X are monovalent organic cations, metal cations, and halide anions, respectively. Previously, the optical and electronic properties of HOIPs have been intensively studied through chemical substitution^2,3^. Being one of the most frequently studied HOIPs, methylammonium lead iodide (MAPbI$_3$) prepared via spin-coating and inverse-temperature crystallization (ITC) method were reported to reach power conversion efficiency exceeding 20%^4–7^. Compared with other HOIPs, MAPbI$_3$ has a small direct band gap of 1.6 eV which is close to the optimum values given by Shockley–Queisser’s and also a high absorption coefficient of $\sim$ 10$^5$ cm$^{-1}$^8,9^. In addition, the effective masses of free electron and hole carriers are relatively low, leading to high electron mobility which is good for high-performance adsorber and electron transporter applications^10–12^.

Prior numerous investigations in electronic and optical properties of hybrid perovskite MAPbI$_3$ (e.g., crystal structure, atomic mixing, spin-orbit coupling, and ion migration) have been presented^13–16^. However, some fundamental knowledge of microscopic behavior in MAPbI$_3$ remains poorly discussed, specifically, the understanding of the orientation of organic MA on the A-site that undergoes the distortion overlap of the inorganic PbI$_6$ octahedra due to the strong hydrogen bonding causing octahedral tilts. The octahedral tilting can be explained by the second-order Jahn-Teller effect as a quantum mechanical theory^17–20^. Despite the fact that the MA interactions play a minor role in the band structure, its interplay has implications more generally in many functional properties (e.g., ferroelectricity, magnetism, and magnetoresistance) in this class of materials^21,22^.

The organic–inorganic interactions (that is, the vdW forces arising from the MA cations and the PbI6 octahedra) can be adjusted by controlling the temperature^23–25^. The fixed orientation of organic MA can be observed at low temperatures, while the free motion of organic MA starts to increase and it is accompanied by straightened Pb-I-Pb bonds at high temperatures^26^. Recently, the blocking of MA reorientational modes is primarily affected by PbI$_6$ octahedral tilting and leads to phase transition in MAPbI$_3$^27^. Applying mechanical pressure also provides an alternative way to induce significant changes in optical and electronic behaviours, including fundamental properties which are rarely observed under ambient conditions^28^. The application of high-pressure technology can cause dramatic changes in both structural and phase transition, including lattice disorder, bulk change, cubic distortion, and octahedral tilting^29–31^. As previously reported, the crystal structure of MAPbI$_3$ at room temperature has been observed as a tetragonal (*I4*/*mcm*) $\beta$-phase and a cubic (*Im*3) $\alpha$-phase at ca. 0.4–2.71 GPa^32–34^. Other observations confirmed that the orthorhombic structure appears at a low pressure of 0.3 GPa then followed by a fully amorphous phase above 3 GPa^35^. Moreover, the metallic phase of MAPbI$_3$ has been observed above 60 GPa^36^. Finally, the pressure can also induce potential physical properties, for instance, bandgap closing, tunable photoluminescence, broadband emission, magnetic ordering, metallization, metal-insulator transition, and carrier lifetime modifications^37–40^.

The crystal structures of MAPbI$_3$ have been investigated using X-ray diffraction under high pressure by several research groups^31,34^. In fact, the position of the lighter atoms such as hydrogen, carbon, and nitrogen are difficult to detect in experiments. On the other hand, Raman spectroscopy, infrared (IR), nuclear magnetic resonance, and neutron scattering have been used to study phonons in this very material as alternatives^40–42^. In addition, Density Functional Theory (DFT) method was used to support the experimental works. Previous work presented that the Raman spectrum range of MAPbI$_3$ observed between 0 and 3200 cm$^{-1}$ and can be classified into three distinct regions: internal vibration of the PbI$_6$ octahedra (0–60 cm$^{-1}$), internal vibration of the organic MA (60–1300 cm$^{-1}$), and vibration of the H-bonds (1300–3100 cm$^{-1}$)^42^. the six additional Raman modes of the H-bonded pairs include the bending of C–H and N–H bonds, associated with the stretching of Pb–I bonds and breathing of PbI$_6$ octahedra^43^. Despite the feasibility of experimental measurements of vibrational modes, a better understanding of pressure-induced vibrational modes, especially in MAPbI$_3$, needs to be developed.

In this work, the MAPbI$_3$ single crystals have been successfully synthesized by using the inverse temperature crystallization (ITC) technique, as reported previously^44^. We tracked how the pressure-induced the Raman modes evolution of MAPbI$_3$ single crystal corresponds to the internal interaction of organic MA and inorganic cation. The diamond anvil cell (DAC) was used to apply the hydrostatic pressure to our samples, while the level of pressure was observed using ruby fluorescence. The Raman spectra of MAPbI$_3$ was measured at various pressure ranging from ambient pressure to high pressure of ca. 5 GPa, in frequencies ranging between 50 and 1500 cm$^{-1}$. To explore the activity of each mode during compression in more detail, we also performed a comprehensive group-theoretical analysis using DFT. By comparing our observed and calculated spectra, we are able to assign a similar sequence of each observed mode under pressure. Owing to the advantages of tracking the vibrational mode evolution under pressure, we gain insight into the relationship between applied pressure with internal vibration that may give a deep understanding for further pressure optimization of MAPbI$_3$ and of other current available perovskites.

## Results

### Raman spectrum at ambient pressure

**Figure 1:**
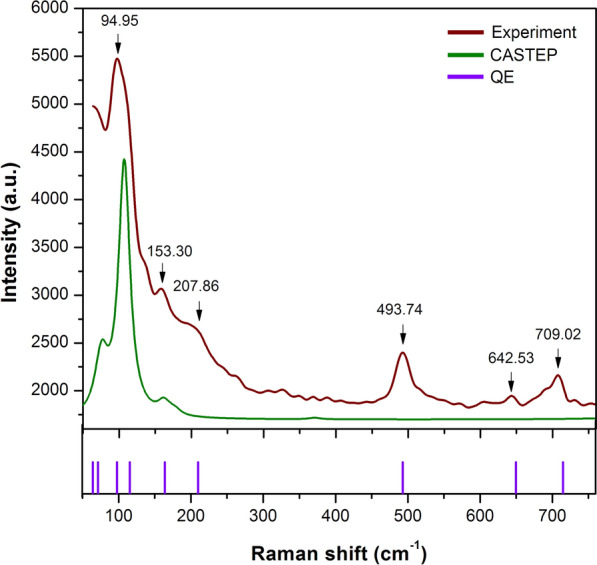
A comparison between observed and calculated Raman spectra of MAPbI$_3$ in the low-frequency range up to 760 cm$^{-1}$. The vertical arrows are guides to the eye and indicate the frequencies of the Raman modes with the highest intensity. The green lines and purple sticks have been obtained from the first-principles calculation through CASTEP codes and QE codes, respectively.

In our work, the Raman spectrum can be neatly separated into two spectral regions: a low-frequency range (60–760 cm$^{-1}$) and a fingerprint frequency range (900–1500 cm$^{-1}$). The Raman modes in the low-frequency range were confirmed by Raman spectroscopy, as reported in Fig. 1. In this region, we observed that the normal modes at low frequency correspond to the internal vibration of the PbI$_6$ octahedra and organic MA cations. The vibrational modes of heavy atoms, i.e. Pb and I, are expected to have lower frequencies. Previous work observed that the peaks of PbI$_6$ framework present first Raman intensity at 41.6 cm$^{-1}$^42^. However, we cannot observe the highest intensity below 60 cm$^{-1}$. In consistent with previous work, the peaks at 153.30 cm$^{-1}$, 207.86 cm$^{-1}$, and 493.74 cm$^{-1}$ are identified as roto-translation organic MA^43^. It was commonly accepted that the several rotational and torsional degrees of organic MA lead to disordered the arrangement of perovskite structure and in turn results in the Rashba spin-orbit coupling^45^. At the higher frequency, we tentatively observed the vibrational modes of rocking and stretching MA at 642.53 cm$^{-1}$ and 709.02 cm$^{-1}$. These two breathing modes are exceedingly sensitive to the H atoms that may affect the electronegativity of the bonding^46^.

In parallel with our observation, the first-principles predictions from CASTEP and QE are also presented Fig. 1. We have optimized the geometry of MAPbI$_3$. According to the QE prediction, we identify at least 4 normal modes as Raman active of PbI$_6$ octahedra and 5 normal modes as Raman active of organic MA across a frequency range between 60 and 760 cm$^{-1}$. These predictions are in apparent agreement with observation especially the calculated vibrational modes of the organic MA. On the contrary, the absent peaks of organic MA, as reported by the CASTEP calculation, are slightly unreliable compared with QE due to the exclusion of spin-orbit interactions. Furthermore, the predicted mode shifts are noticeable at 64.31 cm$^{-1}$, 71.31 cm$^{-1}$, 97.65 cm$^{-1}$, and 115.27 cm$^{-1}$ which are mainly associated with the Pb-I bending and stretching modes.

**Figure 2:**
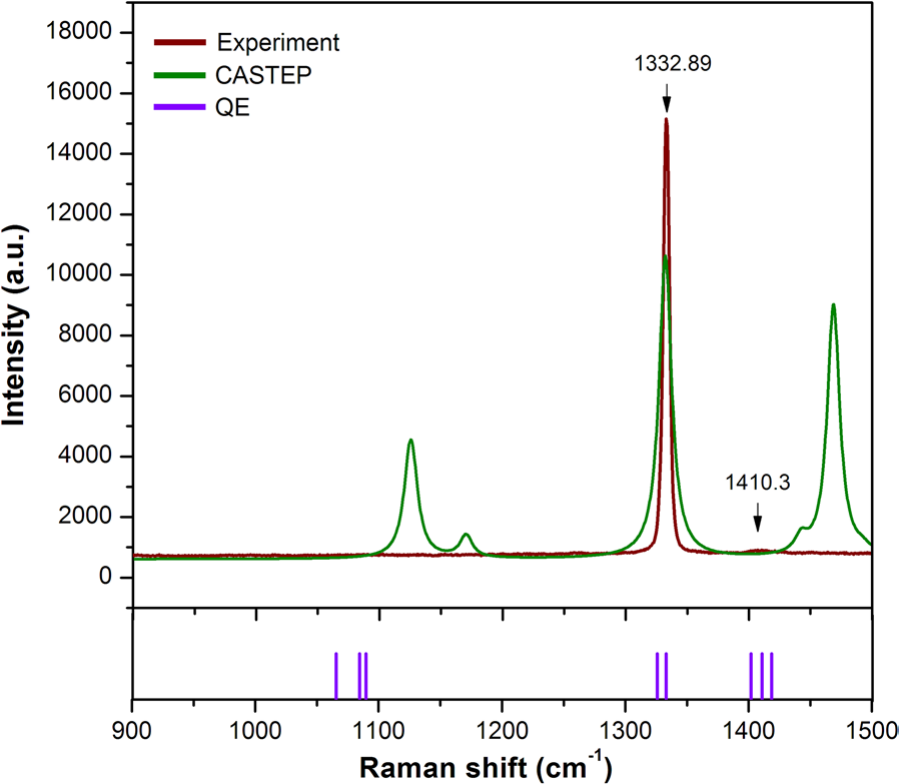
The Raman spectra of MAPbI$_3$ observed at fingerprint frequency range, while their predictions from CASTEP and QE codes are included for comparison. For clarity, the Raman spectra are measured at ambient pressure and room temperature.

We also provide the next Raman spectra from a low frequency of 900 to a high frequency of 1500 cm$^{-1}$ as depicted in Fig. 2. In this frequency range, the modes are mainly associated with hydrogen bonding vibrations. The highest intensity around 1332.89 cm$^{-1}$ and the small feature at 1410.3 cm$^{-1}$ correspond to C–H and N–H bending modes with E-symmetry^42^. However, the observations of Raman modes are quite dissimilar from the predicted results. The observed spectra around 1100 cm$^{-1}$ are imperceptible due to the MA cation being highly sensitive to structural distortions of the PbI$_6$ octahedral framework^47^.

In addition, the computational approach reveals 8 internal vibration modes of organic MA and indicates the character of each mode as CH$_3$–NH$_3$ rocking, C–N stretching, and H-bonds bending. Since organic MA is non-centrosymmetric, the majority of MAPbI$_3$ vibration modes are characterized as Raman active with different relative intensities. By means of the analysis of separate factor groups, the vibrational modes of hydrogen bonding within the organic MA molecule can be expected to be noticeable at 1370, 1403, 1419, 1533, and 1544 cm$^{-1}$^43,48^. This earlier report is inconsistent results obtained from QE codes, whereas the peak positions noticeably shift to a higher frequency in the CASTEP prediction.

**Figure 3:**
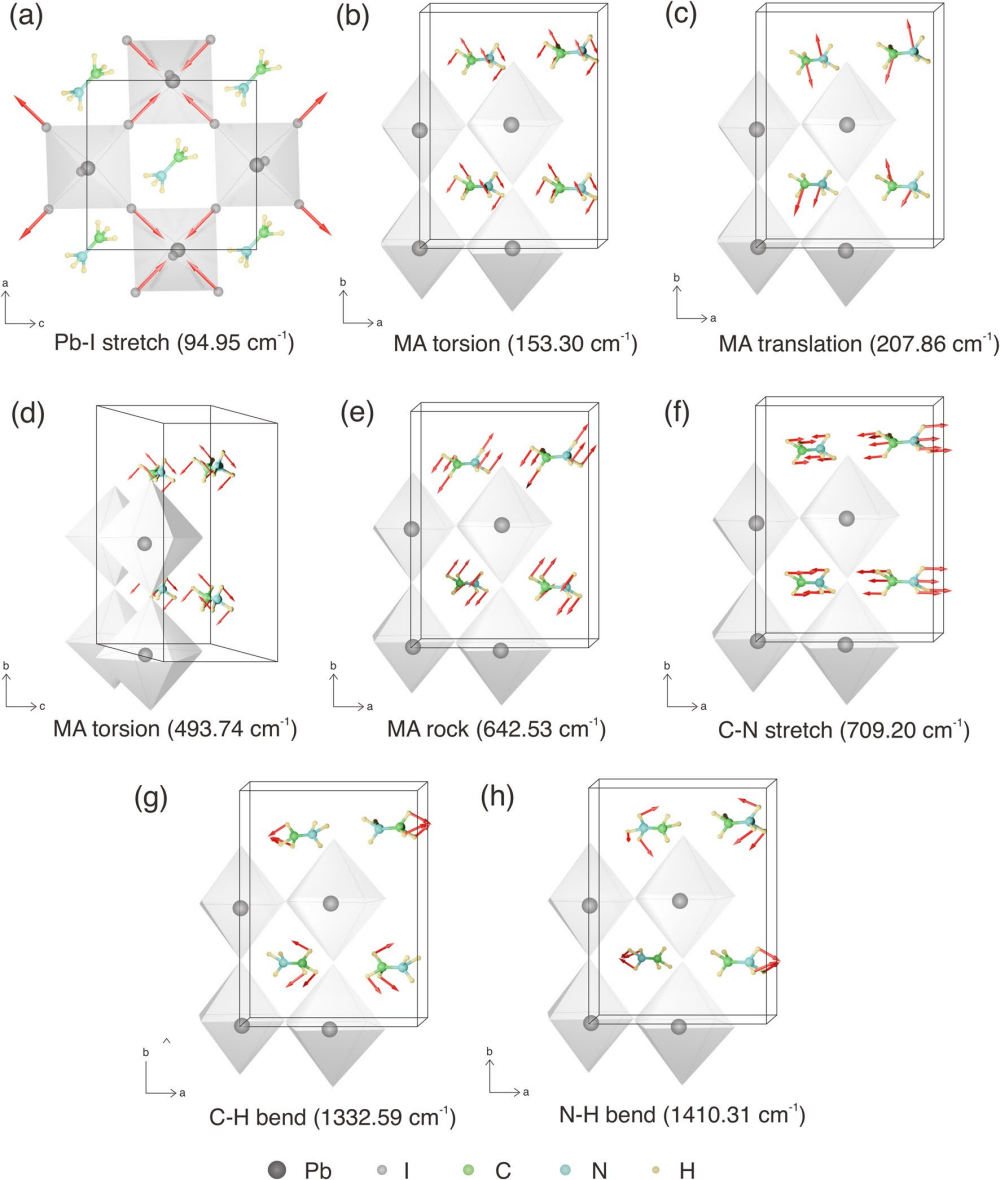
Schematic representations of Raman vibrational modes in MAPbI$_3$. The vibrational modes of octahedral lattice from top view where Pb atoms in black color and I atoms in grey color (**a**); and the internal vibrational modes of organic MA from side view where C, N, and H are in green, blue, and yellow color, respectively (**b**–**h**). The red arrows are the vibrational movements.

The DFT calculations and the observed Raman shifts are comprehensively reported in table 1. A factor group analysis was used to define the symmetry. The calculated normal modes at $\Gamma$ point adopt the following symmetry 2$A_g$ + 8$B_{1g}$ + 5$B_{2g}$ + 2$B_{3g}$ where the g type corresponds to the inversion symmetry. The predicted Raman shifts are in good agreement (within 0.5–1%) with observed Raman peaks and well generate almost all peaks. As mentioned previously, a single peak of PbI$_6$ octahedral framework is observed at 94.95 (cm$^{-1}$) and described as the symmetric Pb–I stretching. In this case, the intensity of vibrational modes with $B_{1g}$ symmetry is high and broad. Hence, the two of Pb–I–Pb bending and asymmetric Pb–I stretching modes cannot be determined. Moreover, the vibrational modes of organic MA, especially at mode numbers 10, 11, and 12 are barely discernible. It can be assumed that the present peak observation of organic MA is assigned as the total vibrations of the entire MAPbI$_3$ crystal. Figure 3 provides the schematic representations of each observed vibrational modes in MAPbI$_3$. The schematic shows atomic motions of organic MA that have the most significant Raman intensity. The vibrational movement of organic MA is most likely due to their high sensitivity to the microenvirontment^49^. Overall, we summarized all possible active modes of MAPbI$_3$ from low to high frequencies as follows: Pb–I stretching modes ($V_3$) of $B_{1g}$ symmetry at 94.95 cm$^{-1}$, MA torsion modes ($V_5$) of $B_{1g}$ symmetry at 153.30 cm$^{-1}$, MA translation/libration modes ($V_6$) of $A_g$ symmetry at 207.86 cm$^{-1}$, MA torsion modes ($V_7$) of $B_{1g}$ symmetry at 493.74 cm$^{-1}$, MA rocking modes ($V_8$) of $B_{1g}$ symmetry at 642.53 cm$^{-1}$, C–N stretching modes ($V_9$) of $B_{2g}$ symmetry at 709.20 cm$^{-1}$, C–H bending ($V_{14}$) and N–H bending modes ($V_{16}$) of $B_g$ symmetry at 1332.59 cm$^{-1}$ and 1410.31 cm$^{-1}$, respectively. For further analyses, we discussed the evolution of its vibrational modes under hydrostatic pressure.

### Pressure-induced Raman modes

**Figure 4:**
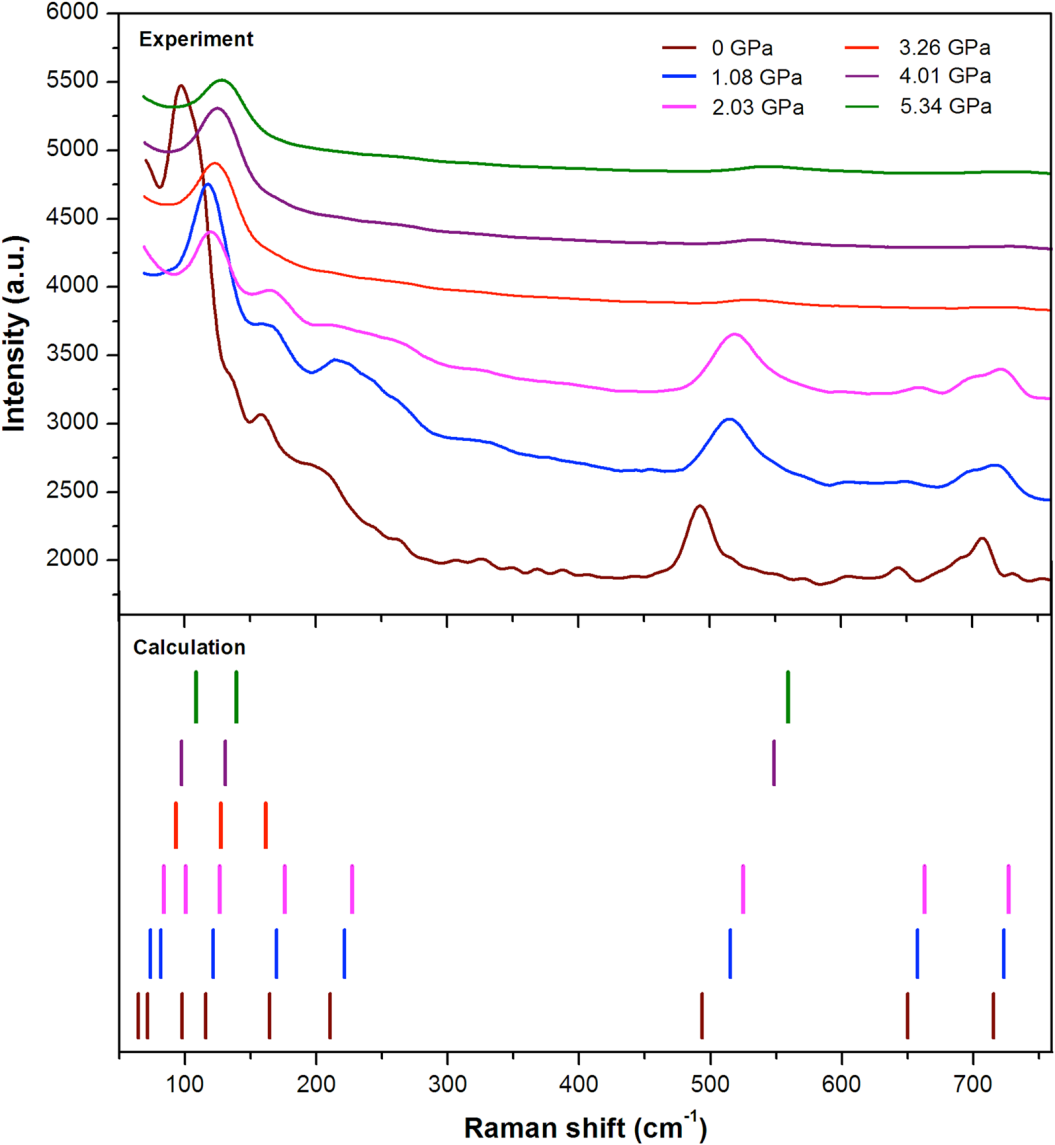
Observed Raman spectra from MAPbI$_3$ upon compression (upper chart) and comparative results of Raman modes by DFT calculation (lower chart).

In this part, we report on the high pressure-induced vibrational evolution in the hybrid perovskite MAPbI$_3$. The phase transition and also the interaction of the organic–inorganic molecule upon hydrostatic pressure were comprehensively studied by using the coupled Raman spectroscopy with the DAC. As shown in Fig. 4, the spectra in the low-frequency range between 60 and 760 cm$^{-1}$ present diverse distributions of peak positions under six steps of applied pressure. At ambient pressure (0 GPa), the first peak around 94 cm$^{-1}$ accounts for the PbI$_6$ octahedral stretch with the highest and largest intensity compared to those of others. This clearly indicates that the vibrational behaviour of PbI$_6$ octahedra causes a significant dynamical disorder in the structural phase at 0 GPa. Moreover, the Raman peak of MA libration has a rather broad range of frequencies (150 cm$^{-1}$ up to 270 cm$^{-1}$). This indicates that the MA cation is freely moving within the perovskite cages at ambient pressure. Previously, the extreme broadening of Raman spectra has been observed at room temperature to be attributable to the full reorientation of MA cation embedded inside the cavity^50^.

The intensity of each peak slightly collapses as the pressure is elevated up to 2.03 GPa, particularly the peaks of $V_3$ below 150 cm$^{-1}$. In this pressure condition can be related to the restraint of Pb–I stretching modes under compression, yet the vibrational modes of $V_5$ and $V_6$ around 160 and 223 cm$^{-1}$, respectively, are still observed. As previously reported, the first two phases were observed below 2.5 GPa through the context of broad Raman spectra along the frequency ranging from 0 to 500 cm$^{-1}$^51^. According to our findings, the frequency of Raman spectra was extended beyond 500 cm$^{-1}$ and we observed that the vibrations of $V_7$, $V_8$, and $V_9$ modes around 515, 660, and 720 cm$^{-1}$, respectively, are still visible at this level of pressure. In principle, there is a small probability of vibrational interaction between PbI$_6$ octahedral frameworks with organic MA in the existence of this phase. Therefore, the organic MA tends to move freely in the voids of inorganic cages with the pressure condition up to 2.03 GPa.

The tremendous spectra change occurs when the sample is compressed beyond 3.26 GPa, where the mostly peaks of organic MA vanish and only a peak of Pb–I stretching mode stands alone. It was clearly that the vibrational motions of organic MA freeze as a result of the shortened PbI$$_6$$ octahedral frameworks under high pressure. The other reason is that their compressed structure causes the organic MA to be trapped in the PbI$$_6$$ octahedral frameworks in random positions and leads to breaking the vibrational modes of Pb–I–Pb bending as indicated by the modes disappearing around 100 cm$$^{-1}$$. The torsional movement of organic MA appears once again at high pressure of 4.01 and 5.34 GPa, even though the peaks are not noticeable. Recently, Jaffe et al. and Marek et al. observed the shortening of Pb–I bonds and increasing angle of Pb–I–Pb as well as induces amorphization of the crystal^13,34^. However, it is possible that the vibrational mode of $$V_3$$ is still detectable under pressure up to 5.34 GPa. After decompression, all peaks return to their initial profiles and shift towards low energy, as can be seen in Fig. S1. This clearly indicates that these phase changes are reversible and all vibrational modes of MA reappear upon decompression. A similar effect has been reported for lead-free double perovskite with the flexible organic cations NH$$_4$$ $$^+$$^52^.

For comparison, we also exploited the DFT method to predict the existence of such modes as seen in a lower chart of Fig. 4. We also predict other vibrational modes of PbI$_3$ at a similar frequency that might give an impact on the peak intensity. In addition, the internal vibrations of organic MA including torsion (153.30 and 493.74 cm$^{-1}$), translation (207.86 cm$^{-1}$), rocking (642.53 cm$^{-1}$), and stretching modes (709.20 cm$^{-1}$) can be clearly distinguished. The dynamical disorder of MAPbI$_3$ at ambient pressure might involve Pb–I and organic MA vibrations^47^. All the modes are still active under pressure of 2.03 GPa. Beyond which, a similar Raman mode is also predicted to adopt a new phase at 3.26 GPa where some modes disappear at frequency around 230 cm$^{-1}$, 550 cm$^{-1}$, 660 cm$^{-1}$, and 740 cm$^{-1}$. Although we cannot observed a new peak beyond 4.01 GPa, we can still predict a new mode around 550 cm$^{-1}$.

**Figure 5:**
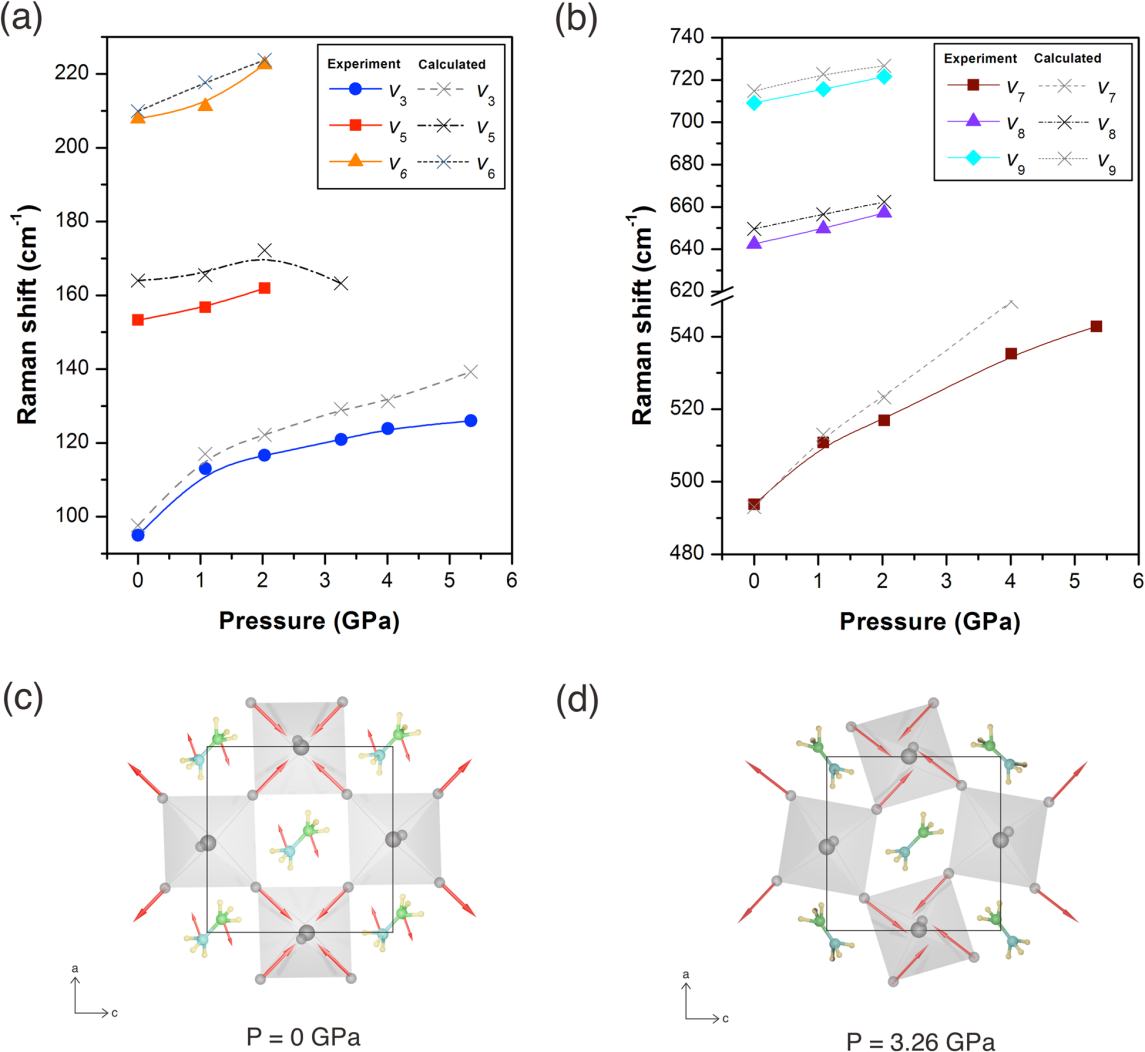
The evolution of Raman shift of MAPbI$_3$ as a function of pressure across frequency range 50–225 cm$^{-1}$ (**a**), 480–740 cm$^{-1}$ (**b**), the schematics of MAPbI$_3$ perovskite illustrate the vibrational motions including PbI$_6$ octahedral stretching modes and internal MA modes under ambient condition (**c**) and hydrostatic pressure at 3.26 GPa (**d**).

Another interesting point concerning the observation of vibrational spectra in MAPbI$_3$ is revealed significant shifts and disappeared over phase transitions under hydrostatic pressure, as provided in Fig. 5a,b. Regarding the trends, we observed the phase evolution under pressure up to 5.34 GPa. As the pressure increases, the Raman peaks shift continuously toward higher energy level, which is attributed to the lattice contraction. Previous work has shown that the volume reduction in MAPbI3 occurs due to octahedral tilting and bond contraction^34^. Our computational calculations further support these findings, revealing that the Pb–I–Pb angle decreases from 155.9 to 151.7 $^{\circ }$C, and the Pb–I bond distance reduces from 3.162 Å to 2.984 Å upon compression. At 0 GPa, the structural fluctuation was expected according to the observed active modes, especially the freely motion (i.e., torsion, translation, rocking, stretching, and bending) of the organic MA and the vibrations of the PbI$_6$ octahedral frameworks as illustrated in Fig. 5c. Likewise, the pressure-induced structural evolution of MAPbI$_3$ has been recently observed by XRD, where their structure is assigned as tetragonal *I*4/*mcm* with a high-level positional disordered of organic MA at room temperature^13,53^. In our case, we can only assume the first phase as the dynamic phase of MAPbI$_3$ since the spectrum data cannot represent the symmetry of the crystal phase. As increasing pressure at 1.08 GPa, The peaks of each vibrational modes including $V_3$, $V_5$, $V_6$, $V_7$, $V_8$, and $V_9$ shift to a higher frequency. Among these modes, the frequencies of $V_3$ and $V_7$ increased more than the other modes at 1.08 GPa. It can be noticed also the intensity of $V_3$ extremely collapses at the distinct frequencies of c.a. 70 cm$^{-1}$. The Raman behavior at this pressure is in agreement with the structural deformation especially in distortion of PbI$_6$ octahedral framework^34,54,55^. However, the distortion does not affect to vibrational properties of organic MA since the vibrational modes of MA remain to exist. Then, the vibrational modes of $V_5$, $V_6$, $V_7$, $V_8$, and $V_9$ are disappear at pressure above 3.26 GPa. Figure 5d illustrates the vibrational motions of organic MA being stopped as a result of the decrease in the unit cell volume when pressurized. In this phase at 3.26 GPa, the compression can affect the vibrational modes of the organic MA where the disappearing peaks ($V_5$ and $V_6$) indicate the reduced motions of organic MA within the voids of the inorganic cage. The new phase, or the static phase, is associated with the saturated intensity around frequency at which the vibration of organic MA takes place. It has been previously suggested that the structural evolution at relatively high pressure as well as temperature entail a very disordered phase and leads to locking the organic MA^34,51^. In addition, the vibrational modes of $V_7$ around 535 cm$^{-1}$ suddenly rises and those of $V_3$ remain as sharp peaks beyond 4 GPa. In other words, the minor changes of the observed spectra are attributable to the distorted static phase under the pressure of 4 GPa. Therefore, our findings indicate that MAPbI$_3$ does not adopt the pressure-induced amorphous phase as previously reported by Francisco–Lopez^51^. Raman observation of H-bond also supported this idea as presented in further discussion.

**Figure 6:**
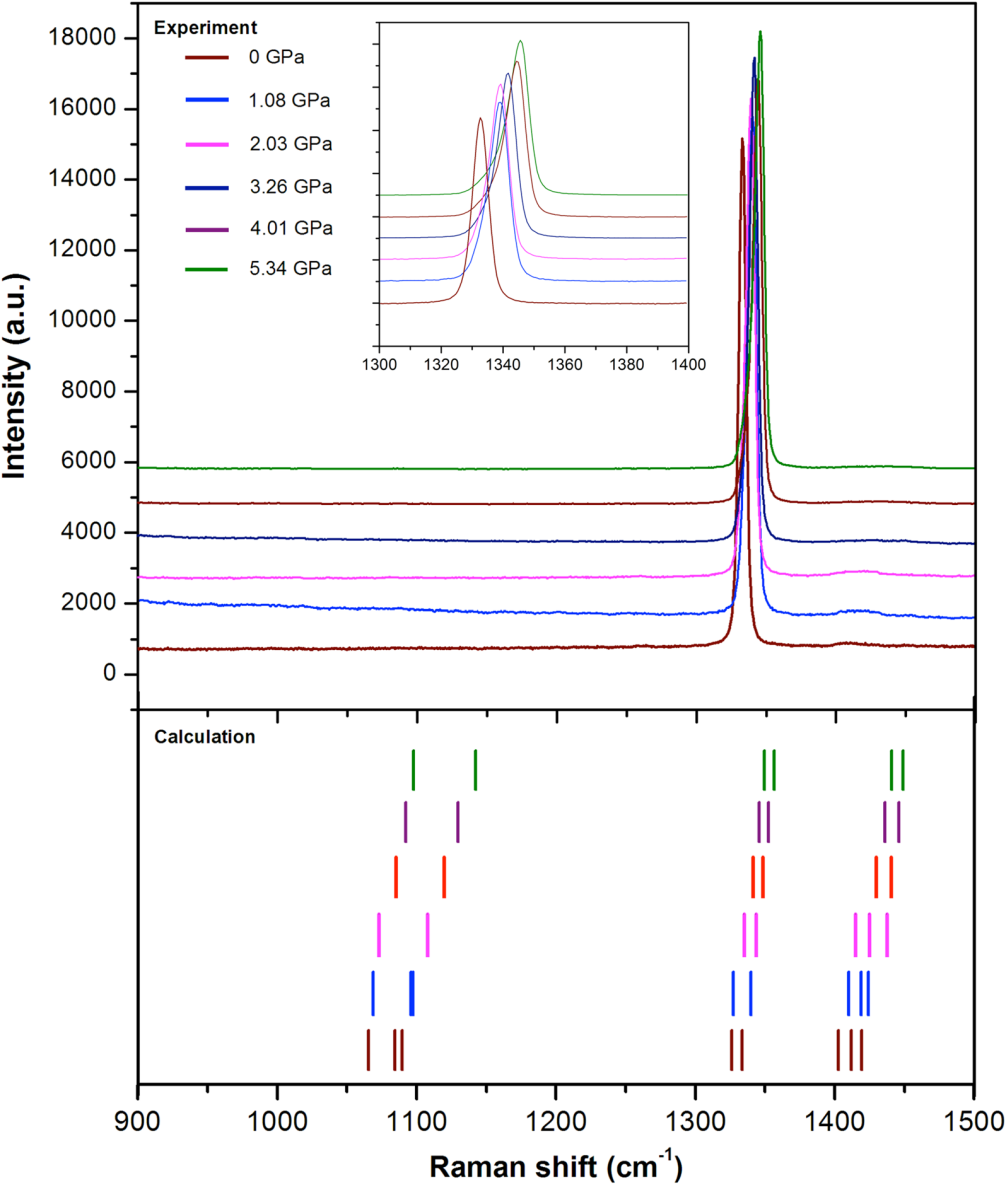
The pressure-induced Raman spectra evolution of MAPbI$_3$ across fingerprint range where observed data shows on an upper chart and calculated data shows on a lower chart. Inset: Raman shift dication.

As seen in Fig. 6, the detail of spectrum in the fingerprint region was observed in DAC under varying pressure. The vibration modes of $V_{14}$ and $V_{16}$ correspond to the well-defined peaks around 1300 and 1400 cm$^{-1}$, respectively, at ambient condition. The observed spectrum intensity of $V_{14}$ is extremely stronger than that of the $V_{14}$ due to the greater bond of C–H, while the peaks splinting is remarkably evident between them. It has been reported that the strengthened molecular hydrogen bonding and motion are affected by the PbI$_6$ octahedra where the increased octahedral tilting exerts a greater interatomic force between hydrogen atoms and iodide ions and eventually leads to an increasing degree of H-bonding^49^. The coupling between organic molecular orientations and the PbI$_6$ octahedral tilting have been acknowledged to be what causes the elongation of the MA molecules and the H bonds^27^.

**Figure 7:**
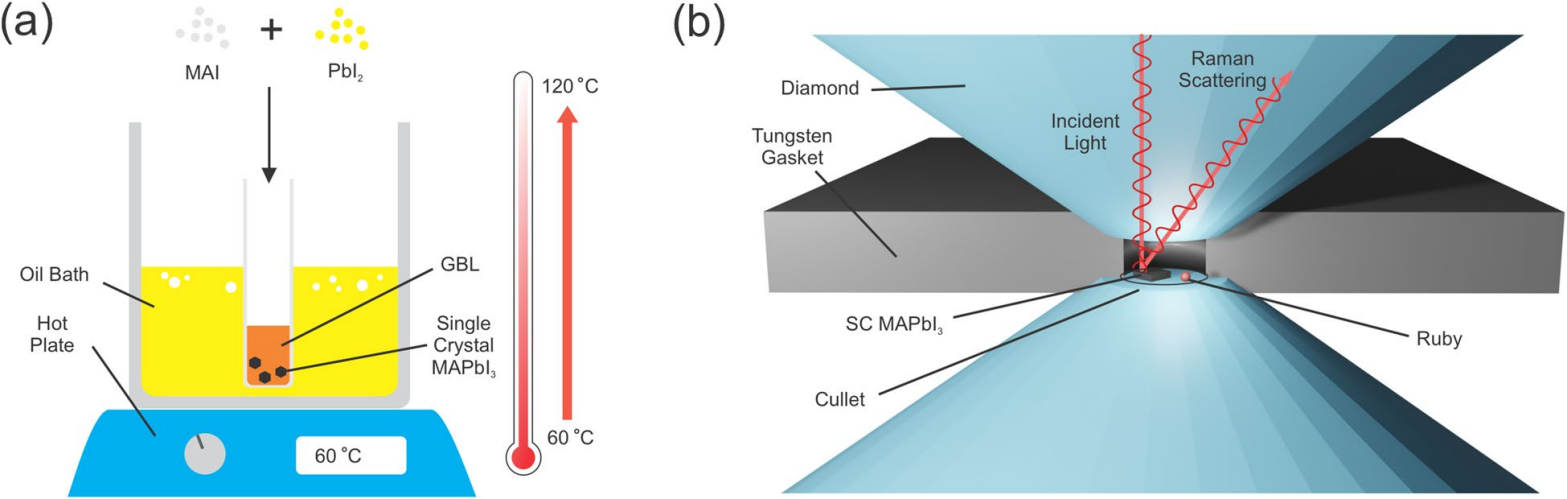
Schematic of experimental approach for inverse temperature crystallization (**a**) and a diamond anvil cells (**b**).

The peaks of $V_{14}$ modes highly shift towards higher energy and yet the intensity exists after compressing. A peak of $V_{14}$ modes slightly shift as pressure increases indicating the decrease in unit volume and reduction of the C–H bending. The peaks of $V_{16}$ modes indicate that the H movement does not interact with the PbI$_6$ octahedral framework. Figure S2 presents the evolution of Raman shifts under pressure which clearly indicates a transition from a dynamic to a static phase. As the pressure reaches 3.26 GPa, the structure enters the static phase. Hence, the peaks of N–H bending modes vanish as the pressure approaches 3.26 GPa. This is probably attributable to the fact that the similar bonding distances of H–I and N–H allow the PbI$_6$ octahedral framework to become tilted and also H atoms more positively charged give high electrostatic interaction^47,56^. Even the rigid body of the MA cation can reside in the anisotropic cubo-octahedra by tilting the PbI$_3$^57^.

**Table 1:** Symmetry, calculated as well as observation Raman shift, and vibrational characters of MAPbI$_3$.

No. mode	Symmetry	Calculated Raman shift (cm$^{-1}$)	Observed Raman shift (cm$^{-1}$)	Character
1	$B_{3g}$	64.31		Pb–I–Pb bend
2	$A_g$	71.31		Pb–I–Pb bend
3	$B_{1g}$	97.65	94.9	Pb–I stretch (sym.)
4	$B_{2g}$	115.27		Pb–I stretch (asym.)
5	$B_{1g}$	163.98	153.3	MA torsion
6	$A_g$	209.83	207.8	Translation/libration
7	$B_{1g}$	493.12	493.7	MA torsion
8	$B_{1g}$	649.64	642.5	MA rock
9	$B_{2g}$	709.77	709.2	C–N stretch
10	$B_{1g}$	1065.62		MA rock
11	$B_{2g}$	1084.54		C–N stretch
12	$B_{1g}$	1089.69		MA rock
13	$B_{1g}$	1325.83		C–H bend (sym.)
14	$B_{2g}$	1333.14	1332.6	C–H bend (asym.)
15	$B_{1g}$	1402.01		N–H bend (sym.)
16	$B_{2g}$	1411.14	1410.3	N–H bend (asym.)
17	$B_{3g}$	1418.68		N–H bend (asym.)

## Conclusions

In summary, we observed the evolution of Raman modes under pressure in organic–inorganic perovskite MAPbI$_3$ along with the DFT calculation. The ITC method was employed in order to obtain single crystal MAPbI$_3$. We tracked the peak positions with frequency ranging between 450 and 1800 cm$^{-1}$ in varying pressure by using DAC which is implemented with Raman spectroscopy. The observed vibrational peaks of MAPbI$_3$ present 8 remarkable peaks and the DFT calculations report 17 modes, e.g., Pb–I stretch, organic MA torsion, libration, rock, and C–H/N–H bend where are existent at ambient pressure. Then, we found at least two major phase changes under pressure up to 5.34 GPa. The transformation from dynamic to static phases occurs at 3.26 GPa and it is followed by a slight change of the Raman spectrum over 4 GPa. The applied pressure leads to the elimination of the internal vibrations of organic MA as a result of the decrease in the PbI$_6$ octahedral stretch. This in turn entails the H-I interactions. Thus, volume reduction under pressure is suggested to play a crucial role in the vibrational reconstructions of these compounds for optoelectronic and ferroelectric applications.

## Materials and methods

### Materials preparation

Methyl Ammonium Iodide (MAI, 99% purity), Lead Iodide (PbI$_2$, 99% purity) powders, and $\gamma$-butyrolactone (GBL, 99% purity) were purchased from Sigma-Aldrich and used as a solvent without further purification. The single crystal of MAPbI$_3$ used in this study was prepared via inverse temperature crystallization (ITC) technique^44,58^. The MAPbI$_3$ was synthesized by reacting MAI CH$_3$NH$_3$I and PbI$_2$ with a molar ratio of 1:1 as depicted in Fig. 7a. The GBL was added dropwise into mixture powders of the MAPbI$_3$ in a vacuum chamber under the nitrogen atmosphere with maintained 10% humidity. The mixture solution was continuously dissolved and stirred on a magnetic hot plate at 70 $^{\circ }$C for 24 h until a yellow saturated solution is visible. The final solution was placed into an oil bath and slowly heated from 60 to 120 $^{\circ }$C with the temperature rate of 10 $^{\circ }$C/hour. Five hours later the large crystals were obtained in black color with a large size of $\sim$ 1 mm. Figure S3 shows a photograph of the top surface of the sample, indicating the crystal sample’s homogeneity on the micrometer scale.

### Experimental methods

By using the diamond anvil cell (DAC), as shown in Fig. 7b, the hydrostatic compression can be applied up to 5.27 GPa. A pair of diamond anvils were placed on the opposite sides of each other, whereas the MAPbI$_3$ single crystal was placed inside the gasket hole together with a ruby sphere and silicone oil as the pressure transmitting medium. In order to calibrate the hydrostatic compression inside DAC, a ruby sphere was placed together with the specimen. The diameters of diamond culet and gasket hole were adjusted to be 100 $\upmu$m and 50 $\upmu$m, respectively, to ensure the attainable pressure of up to 5.27 GPa.

In our work, the Raman spectroscopy (Horiba, iHR 550) was used to determine the vibrational spectra of MAPbI$_3$ using a solid-state laser with 532 nm wavelength. The spectra were detected by a TE-cooled Synapse charge couple device (CCD) system with 1800 lines/mm gratings and can be measured down to 50 cm$^{-1}$ capped by the notch filter. A 532 nm line from a diode laser was carried out for excitation source and kept the lower power density below 15 mW. Previous works have reported a noticeable degradation of hybrid perovskites^59–61^. In order to avoid the thermal effect, the neutral density (ND) filter was used. Figure S4 presents the sample comparison after applied laser irradiation with and without the ND filter. The laser beam is focused onto the samples with a 50$\times$ objective lens employed for both ambient and pressure conditions. The scattered radiation was collected in the back scattering geometry with the acquisition time of 30–60 s and accumulation of 10 times.

### Computational methods

To comprehensively investigate the pressure-induced interaction of the organic–inorganic MAPbI$_3$, we performed the first-principles calculation as implemented in the Quantum ESPRESSO (QE) package. The generalized gradient approximation (GGA) of Perdew-Burke-Ernzerhof (PBE) was employed. In addition, the implementation of projector augmented-wave (PAW) pseudopotentials were generated for atomic compositions as follows: 1 for Pb (5d$^{10}$6s$^2$6p$^2$), 3 for I (5s$^2$5p$^5$), 1 for C (2s$^2$2p$^2$), 1 for N (2s$^2$2p$^3$), and 6 for H (1s$^1$). The plane-wave basis sets with the converged energy cut-offs of 80 Ry were verified for all calculations to optimize the structure geometry of unit cells. The k-point meshes of 8 $\times$ 8 $\times$ 8, as defined by Monkhorst-Packs scheme were gridded for Brillouin zone integration. In order determine the Raman modes of MAPbI$_3$, the phonon calculations were carried out within Density Functional Perturbation Theory (DPFT) using the same set of DFT parameters^62^. At first, we determined the dynamical matrices at $\Gamma$ point with the phonon threshold of 1$\times$10$^{-15}$ meV. Then, the eigenmodes and frequencies were obtained from calculating the interatomic force constant in real space. We also performed the DFT calculation based on the Cambridge Serial Total Energy Package (CASTEP) code as a comparison^63^.

### Supplementary Information

Supplementary Figures.

## Data Availability

The data that support the findings of this study are available from the corresponding author upon reasonable request.
