# Supplementary material for: Phase transformations and vibrational properties of hybrid organic–inorganic perovskite MAPbI3 bulk at high pressure
Source: Sci Rep. 2023 Oct 6;13:16854. doi: 10.1038/s41598-023-43020-1 (PMC10558557; doi:10.1038/s41598-023-43020-1)
Supplement: Supplementary file 1 — Supplementary Figures. [file 41598_2023_43020_MOESM1_ESM.pdf]

Phase Transformations and Vibrational Properties of Hybrid Organic-Inorganic Perovskite $MAPbI_3$ Bulk at High Pressure

Ardimas,
Teerachote Pakornchote,
Wiwittawin Sukmas,
Sojiphong Chatraphorn,
Stewart J. Clark,
Thiti Bovornratanaraks

August 2022

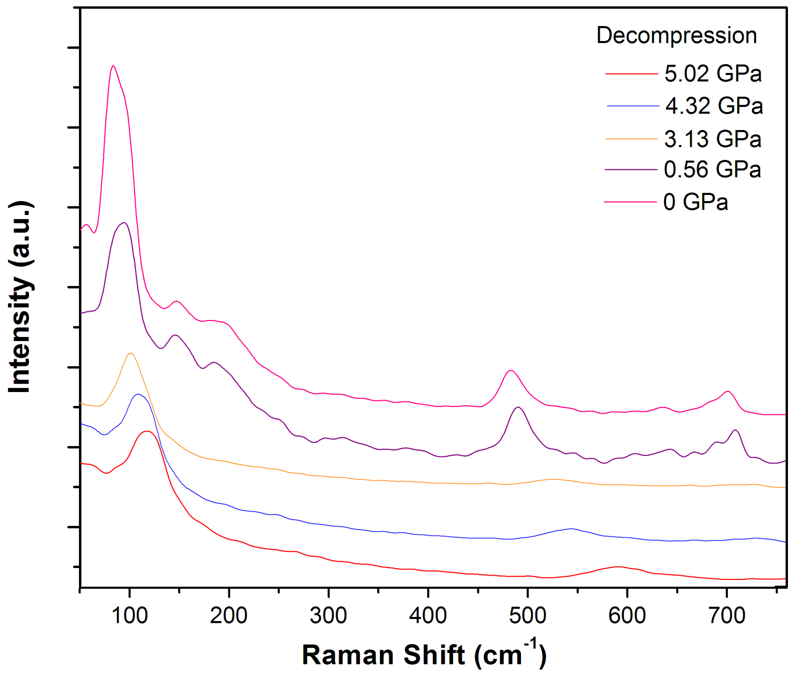

Figure S1: Raman spectra evolution of $MAPbI_3$ during decompression from high pressure to ambient pressure.

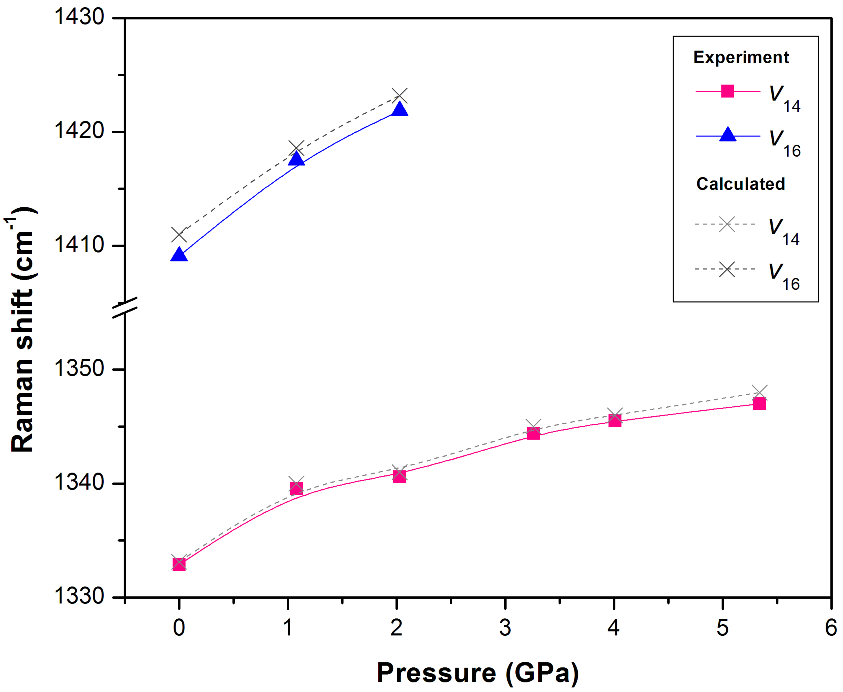

Figure S2: The evolution of Raman shift of $MAPbI_3$ as a function of pressure across frequency range 1330–1430 cm^{-1}.

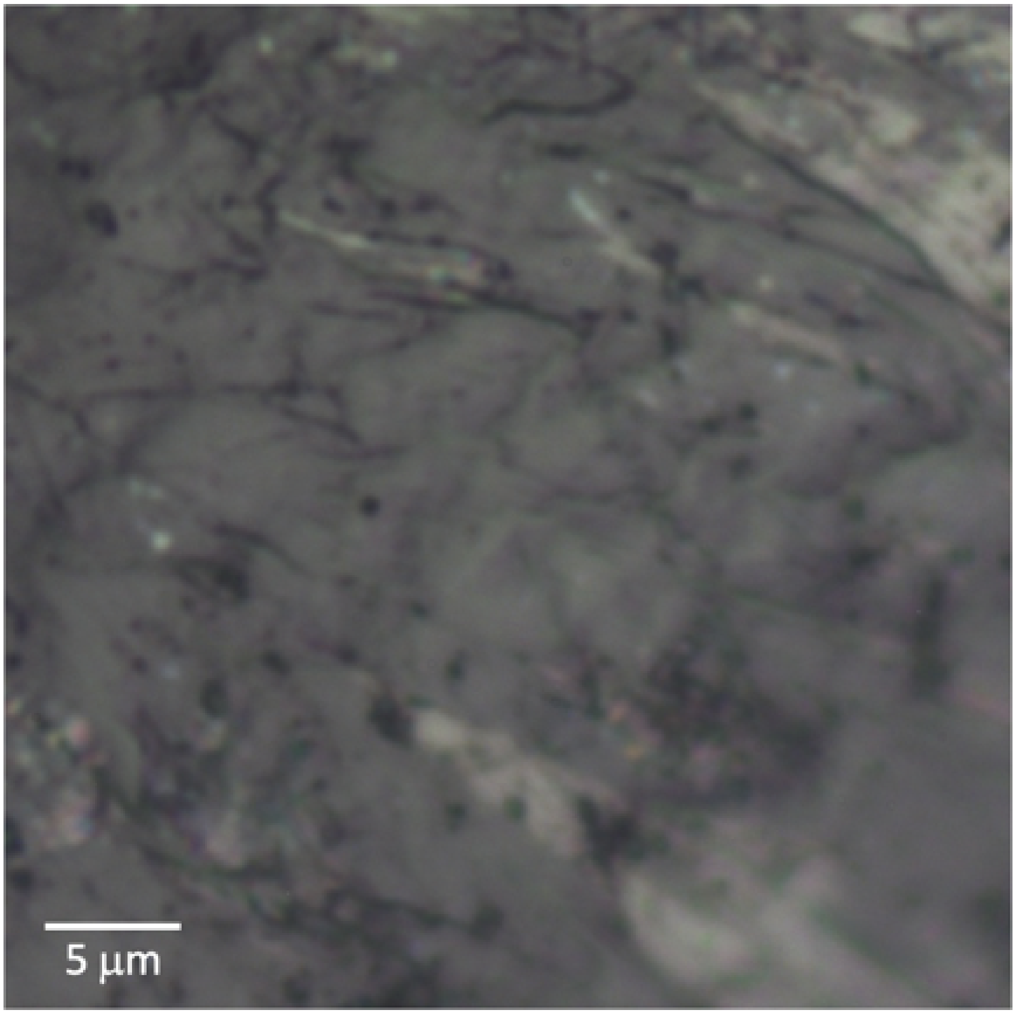

Figure S3: A photograph of $MAPbI_3$ crystal bulk using Raman spectroscopy.

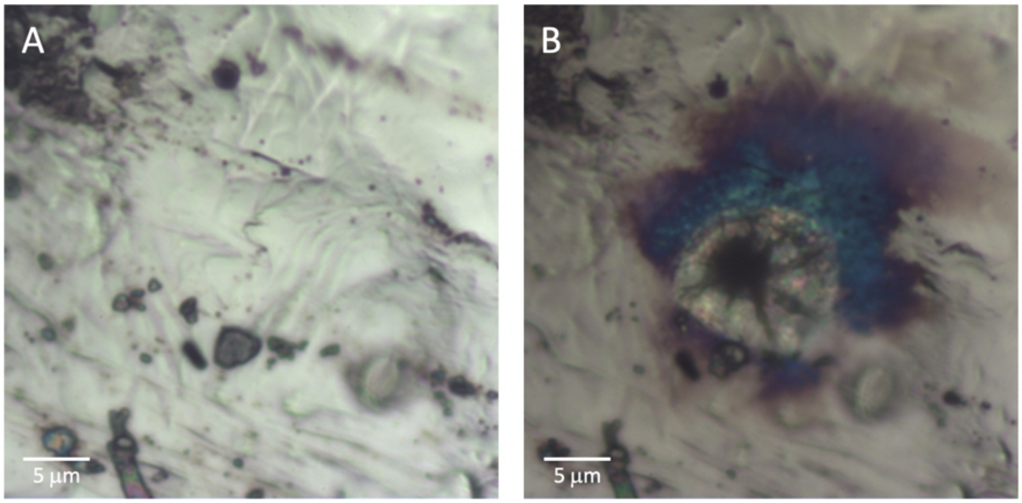

Figure S4: Photograph comparison $MAPbI_3$ crystal bulk with ND filter(a) and without ND filter(b)
